# Perception of footwear comfort and its relationship with the foot structure among youngest-old women and men

**DOI:** 10.7717/peerj.12385

**Published:** 2021-10-19

**Authors:** Ewa Puszczalowska-Lizis, Karolina Koziol, Jaroslaw Omorczyk

**Affiliations:** 1 Medical College, Institute of Health Sciences, University of Rzeszow, Rzeszow, Poland; 2 Health Center Tuchow, Tuchow, Poland; 3 Faculty of Physical Education and Sport, Institute of Sport, University of Physical Education in Krakow, Krakow, Poland

**Keywords:** Public health, Human foot, Footwear comfort, Health policy

## Abstract

**Background:**

Adequate footwear comfort and functionality are important regardless of age, but they become particularly important in the youngest-old women and men, mainly due to the fact that this age range is the initial period of old age with changes in shoe preferences. The aim of this study was to assess the perception of footwear comfort and its relationship with the feet structure in youngest-old women and men.

**Methods:**

The cross-sectional study covered community dwellers living on their own aged 65–74 years (50 women; 50 men). The feet characteristics were measured using the CQ-ST podoscope (Electronic System, Ltd, EU), and the perception of footwear comfort was assessed with a visual analogue scale. The assessment took into account gender-specific footwear of a certain brand (Befado Dr orto).

**Results:**

Statistically significant intergender differences were observed in the perception of footwear comfort with respect to the shoe heel width (*p* = 0.022), the arch height (*p* = 0.013), the overall comfort (*p* = 0.049) and the material properties of the footwear (*p* = 0.017). In women, there were statistically significant positive relationships among the heel angle (*γ*) and the perception of footwear comfort in terms of heel cushioning (*p* = 0.021), forefoot cushioning (*p* = 0.015), arch height (*p* = 0.029). In men, there was a statistically significant negative relationship of the left foot Clarke’s angle with the heel height (*p* = 0.043), and a positive relationship between the right foot width and the arch height (*p* = 0.044).

**Conclusions:**

Youngest-old women, compared to men of the same age range, have a higher perception of shoe comfort in terms of the shoe heel width, the arch height, the overall comfort of the footwear and the material properties of the footwear. The appropriate profile and construction of the shoe allows for an increase in the contact surface of the foot with the shoe, hence the improvement in the perception of footwear comfort in people with lowered arch or widened forefoot.

## Introduction

The foot of an aging person is subjected to changes such as joint stiffness, deformation, skin thinning, loss of fat lining, and reduction of muscle mass. The loss of strength and flexibility of the muscle tendons and joint ligaments has the effect of increasing the length and width of the feet, which means that with age seniors need shoes that are wider and/or longer than in the past (Mickle et al., 2010; Ansuategui Echeita et al., 2016). Improperly selected footwear may intensify age-related foot changes, be a direct cause of pain, health disorders of the feet and higher parts of the kinematic chain, and reduce the comfort of locomotion and the ability to undertake everyday life activities (Jiménez-Ormeño et al., 2011; Ikpeze, Omar & Elfar, 2015; Hinojo-Pérez et al., 2016; Jellema et al., 2019). It may cause pressure on some parts of the foot, change the distribution of pressure forces on the foot, thereby increasing the anteroposterior and mediolateral tilts of the body’s centre of gravity, which, combined with weakening posture control mechanisms, predisposes to loss of balance and, consequently, to falls (Hatton et al., 2013; Ikpeze, Omar & Elfar, 2015; O’Rourke et al., 2020). The use of excessively worn or slippery footwear has a negative effect, as well as its improper construction, *e.g.*, wrong height of the heel or upper, heel stiffening (Saghazadeh, Kitano & Okura, 2015). On the one hand, the choice of footwear in the case of seniors stems from economic reasons, which depend on the amount of income from retirement or disability pensions, as well as on savings accumulated during life—the so-called old age security (Jenkins & Knopf-Amelung, 2013; Kim & Do, 2019). On the other hand, some people buy shoes based on fashion or aesthetics rather than comfort. This is especially true about women who have habits from the past when they wore narrow-toed shoes with high heels (Cronin, Barrett & Carty, 2012).

Women’s feet are characterized by a more delicate structure, both in relation to the size of the bone elements and the strength of active-passive stabilizers. For example, they have a smaller and more rounded head of the first metatarsal bone, which, in connection with the muscle strength declining with age, may reduce the stability of the metatarsophalangeal joints. It is also worth emphasizing that after the age of 50, due to the deficiency of sex steroids, the bones of female feet are more prone to osteoporotic changes (Alswat, 2017; Anderson, Williams & Nester, 2017; Cauley, 2018; Wilson, De Groote & Humphrey, 2020). So they may react differently in contact with footwear than men’s feet (Van der Zwaard et al., 2014; Puszczalowska-Lizis et al., 2019).

Caring for the comfort and functionality of footwear is important regardless of age, but in the case of youngest-old it becomes particularly important, mainly due to the fact that this age range is the initial period of old age, initiating changes in shoe preferences. The purchase of footwear cannot be guided solely by aesthetic considerations. The basic features that lead to a decision about purchase should include its functionality and prophylaxis and health-promoting properties (Branthwaite, Chockalingam & Greenhalgh, 2013; Menz et al., 2015; López-López et al., 2016; Hurst et al., 2017). During this period, it is important to neutralize the effects of progressive involution changes in order to ensure the comfort and quality of life, as well as to maintain optimal functional fitness for the next stages of old age. Guidozzi (2017) stressed that as older community-dwelling women and men are the most active part of the older population, they are subject to environment-dependent risk factors for foot problems including those resulting from inappropriate footwear.

The presented facts became a direct reason for undertaking the topic of the study, the aim of which was focused on the assessment of the perception of footwear comfort and its relationship with the foot structure in youngest-old women and men. The following research questions were addressed:

1. Do the youngest-old demonstrate differences between genders in selected features of foot morphology?
2. Do the youngest-old show any inter-gender differences in the perception of shoe comfort?
3. What are the relationships between the subjective perception of footwear comfort and the features of foot structure in youngest-old women and men?

## Material & Methods

### Participants

We examined free-living community dwellers aged 65–74 years (50 women; 50 men). The subjects were randomly selected by means of dependent simple sampling (without return) from among those who completed 65 years of age, were at a purpose-built, housing estate for the seniors. The inclusion criteria were as follows: age between 65 and 74 (youngest-old); living at a purpose-built, housing estate for the seniors; having right upper limb dominating (based on the Waterloo Handedness and Footedness Questionnaire–Revised (Muddle et al., 2017); physical fitness allowing walking without orthopaedic aids; able to stand on the podoscope without assistance; wearing shoes specific for gender of a particular brand (Befado Dr orto) for seven days preceding the survey, for minimum of 7 h a day; written informed consent to participate in the study. People with severe cognitive impairment, with neurological diseases, with any form of lower amputations, or using first aid dressings or orthoses, or severe dependency were duly excluded from the study. The reasons for the exclusion from the study were also recent injuries in any part of the lower extremity or any disorders in foot bones, as well as occurrence of wounds, ulceration of the feet and painful deformation of the fingers (such as hammer toes).

Sociodemographic and clinical characteristics of the study group is presented in Table 1.

**Table 1:** Sociodemographic and clinical characteristics of the study subjects.

Variable	Women (*n* = 50)	Men (*n* = 50)	Statistics
Age (years), mean ± SD	69.62 ± 3.29	69.82 ± 3.01	*Z* = −0.38; *p* = 0.697
Body mass [kg], mean ± SD	71.39 ± 11.13	82.35 ± 10.89	*Z* = −4.53; *p* < 0.001^*^
Body height [cm], mean ± SD	162.16 ± 4.46	172.98 ± 5.76	*Z* = −7.62; *p* < 0.001^*^
BMI, mean ± SD	27.04 ± 4.16	27.48 ± 3.27	*Z* = −0.51; *p* = 0.609
Body built, n (%)			
Underweight	2 (4.0)	0 (0.0)	χ^2^(3) = 2.07; *p* = 0.557
Normal weight	13 (26.0)	14 (28.0)	
Overweight	22 (44.0)	22 (44.0)	
Obesity	13(26.0)	14 (28.0)	
Marital status, n (%)			
Single	1 (2.0)	7 (14.0)	χ^2^(3) = 10.89; *p* = 0.012^*^
Married	30 (60.0)	23 (46.0)	
Divorced	0 (0.0)	5 (10.0)	
Widowed	19 (38.0)	15 (30.0)	

**Notes.**

mean, arithmetical average value; SD,standard deviation; Z, value of the Mann–Whitney U test statistic; *n*, number of subjects; %, percent of subjects; χ^2^, value of the Chi-square test statistic; *p*, probability value.

* α = 0.05.

### Design

**Figure 1:**
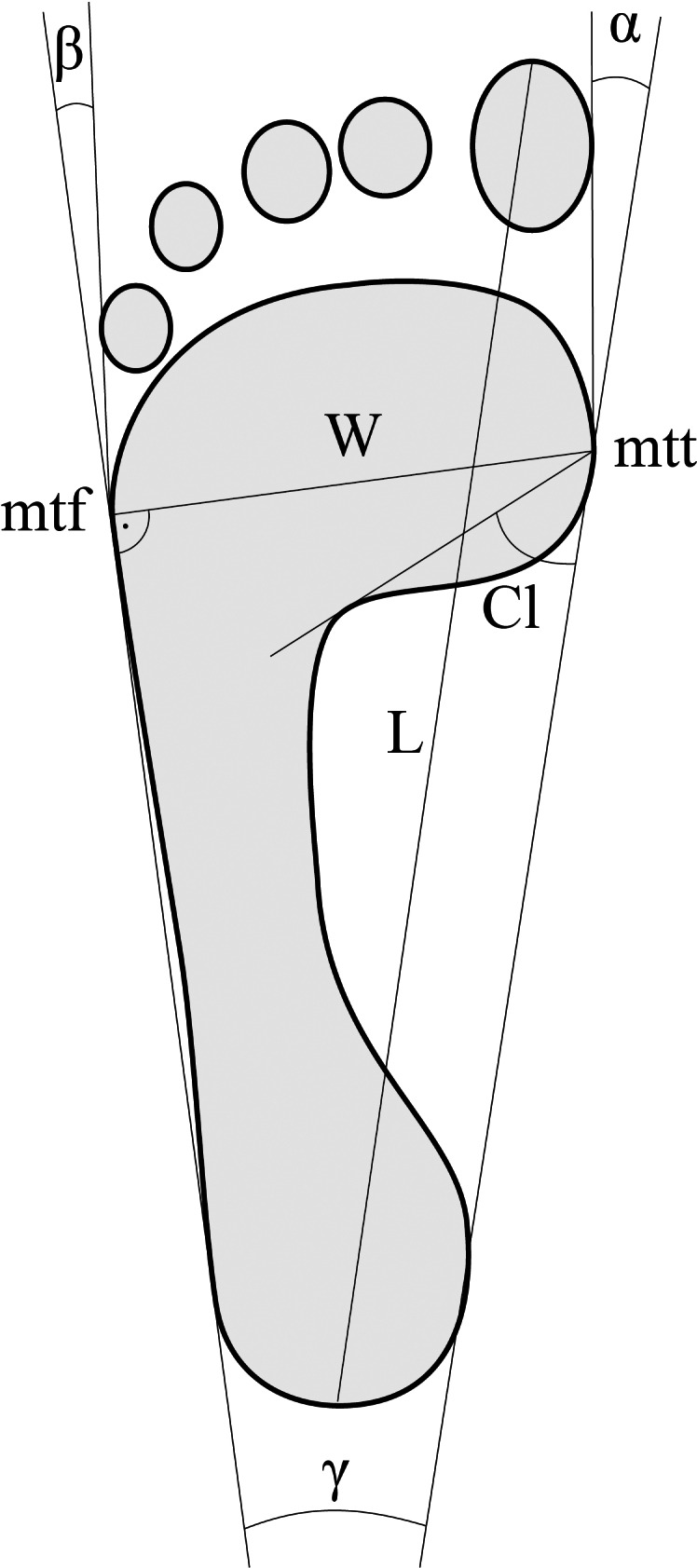
The manner of determining the foot structure indices. L, foot length; W, foot width; mtt, metatarsale tibiale point; mtf, metatarsale fibulare point; Cl, Clarke’s angle; γ, heel angle; α, hallux valgus angle; β, angle of the varus deformity of the 5th digit.

Anthropometric measurements of the body mass (using a SECA 635 medical scales, Seca, Ltd, Germany) and height (using a SECA 264 stadiometer, Seca, Ltd, Germany) were taken. Based on collected data the Body Mass Index (BMI) was calculated. Then, the respondents filled in a form containing questions about age, gender and marital status. The following foot characteristics were measured using the CQ-ST podoscope (Electronic System, Ltd, EU): foot length (L), foot width (W), Clarke’s angle (Cl); heel angle (γ); hallux valgus angle (α) the angle of the varus deformity of the 5th digit (β) (Pita-Fernández et al., 2015; Puszczalowska-Lizis et al., 2017). The procedures for determining these indices are shown in Fig. 1.

The study was cross-sectional. The measurements were conducted in April 2021, in the seniors clubs. The subjects did not undertake physical activity during the 12 h preceding the measurements. To ensure integrity of the research, all tests were performed in the morning, by means of the same measuring instruments that were operated by the authors. During the podoscopic examination seniors were without footwear and socks, wearing sportswear. The participants had their feet disinfected and dried prior to measurement. After getting off the podoscope, they put on socks and shoes and proceeded to the assessment of footwear comfort perception. Before testing the perception of footwear comfort, participants wore the tested footwear for 20 min, then covered a distance of 10 m at their comfortable walking speed.

A visual analogue scale (VAS) was used to assess perception of footwear comfort, which is considered a reliable measure of subjective footwear perception, as ICC=0,799 (Mündermann et al., 2002).

Seniors rated the following aspects of the footwear related to its perceived comfort:

1. shoe length–length of the shoe;
2. shoe forefoot width–width of the shoe in the forefoot region;
3. shoe heel width–width of the shoe in the heel region;
4. heel height–height at which the hindfoot is raised in relation to the forefoot;
5. heel cushioning–softness/hardness of the midsole in the heel region;
6. forefoot cushioning–softness/hardness of the midsole in the forefoot region;
7. arch height–medial arch height of the insole;
8. mediolateral control–position of the foot controlled by the shoe;
9. overall comfort—overall impression of the shoe (Mündermann et al., 2002; Dinato et al., 2015).

Additionally, one more structural element of the footwear was assessed, which influences comfort perception, such as “material properties of the footwear”, which depend on the quality/number of fabrics used in the design and the type of sewing.

The comfort scale used in the study was 100 mm long with “not comfortable at all” (0 comfort points) marked at the left end and “most comfortable” (10 comfort points) marked at the right end. The respondents assessed the comfort of the footwear jointly, both in relation to the right and left feet (Mündermann et al., 2002).

The assessment took into account footwear specific for gender of a certain brand (Befado Dr orto) that subjects wore for seven days prior to the study, for minimum of 7 h a day. These were the participants’ own shoes, purchased by them, in a size adequate to the length of the feet. Before starting the testing, the investigators additionally checked the suitability of the shoes to the tested feet while participants stood in an even weight-bearing position. A shoe was considered fit when the toes were free to move and not locked in the forefoot, and the heel was securely positioned at the heel counter. The credibility of seniors’ participation in the 7-day shoe testing process was recognized on the basis of their declaration. Additionally, during the tests, the wear condition of the shoes was inspected. All respondents wore the same model of footwear, but shoes dedicated for women were beige (catalogue no. 036D005), and men’s shoes–black (catalogue no. 036M007). The choice of this model of footwear was dictated by a relatively low price and high quality, especially in the context of functional and health features. These shoes are characterized by a high, wide shoe forefoot width, a stiffened shoe heel, a profiled insole and a non-slip, shock-absorbing sole. The upper is made of a flexible, stretchable material, and the velcro fastening facilitates flexing and adjusting to the foot’s thickness. The lining is made of a soft “Silber (Ag)” fabric made in a special technology based on silver ions.

The perception of footwear comfort was assessed by the respondents in the presence of the examiner, after obtaining detailed explanations about the assessed parts/aspects of the shoes. The subjects received detailed instructions on how to mark the results on a visual analogue scale. If necessary, additional detailed explanations were provided.

The Bioethics Committee of the University of Rzeszow approved the study (Approval Ref. No. 4/04/2020). All procedures were carried in line with the Declaration of Helsinki. All participants gave their written informed consent to participate in the study protocol.

### Analysis

Consistency of pertinent variables with reference values in normal distribution was verified by means of the Shapiro–Wilk test. The dependences between qualitative data, defining body composition, and marital status were tested with the Pearson Chi-square test. The assessment of inter-gender differences in the values of features determining age, body build, foot structure, as well as the perception of footwear comfort was made using the Mann–Whitney U test. Spearman’s rank correlation was used to analyze the relationship between the foot structure features and the subjective assessment of footwear comfort. The statistical power analysis of our study was 0.88, which is above the lowest recommended power of 0.80 (Cohen, 2013). The statistical significance was set at *p* < 0.05. The Stat Soft STATISTICA application (version 13.1) was used to process all test results.

## Results

Data in Table 2 indicate statistically significant differentiation in terms of the foot length (right foot: *p* < 0.001; left foot: *p* < 0.001) and width (right foot: *p* < 0.001; left foot: *p* < 0.001) in the studied women and men. Women’s feet were shorter and narrower. In the case of the remaining indices, no statistically significant inter-gender differences were found.

**Table 2:** Comparison of the foot structure features of the studied women and men.

Variable		Women		Men		Mann–Whitney U test
		Mean ± SD	Me	Mean ± SD	Me	
Foot length [cm]	rf	22.36 ± 1.12	22.55	24.40 ± 1.21	24.25	*Z* = −6.88; *p* < 0.001^*^
	lf	22.38 ± 1.10	22.50	24.42 ± 1.18	24.45	*Z* = −6.92; *p* < 0.001^*^
Foot width [cm]	rf	8.63 ± 0.56	8.70	9.22 ± 0.65	9.20	*Z* = −4.81; *p* < 0.001^*^
	lf	8.64 ± 0.59	8.60	9.23 ± 0.64	9.20	*Z* = −4.55; *p* < 0.001^*^
Clarke’s angle [°]	rf	32.86 ± 8.45	33.00	33.42 ± 9.69	35.50	*Z* = −0.54;*p* = 0.592
	lf	32.48 ± 7.51	32.00	33.24 ± 9.03	35.00	*Z* = −0.98; *p* = 0.328
Heel angle (γ) [°]	rf	16.14 ± 2.17	15.50	15.92 ± 1.76	15.00	*Z* = 0.32; *p* = 0.746
	lf	16.26 ± 2.40	15.00	15.68 ± 1.46	15.00	*Z* = 0.67; *p* = 0.504
Hallux valgus angle (α) [°]	rf	10.18 ± 7.45	9.00	9.84 ± 7.15	9.00	*Z* = 0.09; *p* = 0.931
	lf	10.38 ± 7.38	10.00	10.22 ± 6.05	9.00	*Z* = −0.10; *p* = 0.923
Angle of the varus deformity of the 5th digit [°]	rf	16.60 ± 6.52	17.00	16.26 ± 5.74	15.00	*Z* = 0.22; *p* = 0.822
	lf	15.04 ± 6.99	15.00	15.30 ± 5.63	15.00	*Z* =−0.11; *p* = 0.909

**Notes.**

rf, right foot; lf, left foot; Mean, arithmetical average value; SD, standard deviation; Me median; *Z*, value of the Mann–Whitney U test statistic; *p*, probability value.

* α = 0.05.

Comparison of the variables determining the perception of footwear comfort showed statistically significant inter-gender differences in the shoe heel width (*p* = 0.022), the arch height (*p* = 0.013), the overall comfort of the footwear (*p* = 0.049) and the material properties of the footwear (*p* = 0.017). Women found the footwear more comfortable (Table 3).

**Table 3:** Comparison of the perception of footwear comfort of the studied women and men.

Variable	Women		Men		Mann–Whitney U test
	Mean ± SD	Me	Mean ± SD	Me	
Shoe length	8.68 ± 1.08	9.00	8.32 ± 1.60	8.00	*Z* = 0.78; *p* = 0.438
Shoe forefoot width	8.44 ± 1.61	9.00	8.30 ± 1.46	9.00	*Z* = 0.87; *p* = 0.387
Shoe heel width	7.20 ± 1.14	7.00	6.60 ± 1.29	7.00	*Z* = 2.29; *p* = 0.022^*^
Heel height	7.42 ± 0.97	7.00	6.94 ± 1.48	7.00	*Z* = 1.46; *p* = 0.143
Heel cushioning	7.00 ± 1.12	7.00	6.60 ± 1.32	7.00	*Z* = 1.84; *p* = 0.066
Forefoot cushioning	7.48 ± 1.09	7.50	7.16 ± 1.36	7.00	*Z* = 0.81; *p* = 0.416
Arch height	7.52 ± 1.05	8.00	6.84 ± 1.22	7.00	*Z* = 2.50; *p* = 0.013^*^
Mediolateral control	7.06 ± 1.00	7.00	6.86 ± 1.28	7.00	*Z* = 0.46; *p* = 0.644
Overall comfort	8.96 ± 1.11	9.00	8.38 ± 1.50	9.00	*Z* = 1.96; *p* = 0.049^*^
Material properties of footwear	8.16 ± 1.09	8.00	7.44 ± 1.43	7.50	*Z* = 2.40; *p* = 0.017^*^

**Notes.**

Mean, arithmetical average value; SD, standard deviation; Me, median; *Z*, value of the Mann–Whitney U test statistic; *p*, probability value.

* α = 0.05.

Data in Table 4 indicate in women statistically significant relationships among the heel angle (γ) and the perception of footwear comfort in terms of heel cushioning (*R* = 0.33; *p* = 0.021), forefoot cushioning (*R* = 0.34; *p* = 0.015) and the arch height (*R* = 0.31; *p* = 0.029). The positive direction of the abovementioned relationships indicates that higher values of the heel angle are accompanied by a better perception of footwear comfort.

**Table 4:** Relationships of foot structure features with subjective assessment of footwear comfort in women.

Variable		L_*r*_	L_*l*_	W_*r*_	W_*l*_	Cl_*r*_	Cl_*l*_	γ_*r*_	γ_*l*_	α_*r*_	α_*l*_	β_*r*_	β_*l*_
Shoe length	R p	0.14 0.338	0.15 0.299	−0.01 0.953	−0.04 0.776	−0.25 0.085	−0.27 0.055	−0.13 0.381	0.05 0.707	−0.05 0.733	−0.10 0.484	0.08 0.578	0.03 0.856
Shoe forefoot width	R p	0.04 0.784	0.03 0.811	0.02 0.913	−0.05 0.749	−0.14 0.338	−0.18 0.206	−0.15 0.306	−0.03 0.827	−0.03 0.848	−0.06 0.673	0.07 0.606	−0.04 0.809
Shoe heel width	R p	0.03 0.815	0.02 0.901	0.10 0.476	0.08 0.585	−0.08 0.603	0.02 0.899	0.20 0.170	0.19 0.183	0.04 0.765	0.03 0.822	0.09 0.518	−0.11 0.463
Heel height	R p	0.01 0.921	0.03 0.834	0.09 0.557	0.05 0.730	−0.25 0.084	−0.24 0.095	−0.08 0.583	−0.06 0.672	0.04 0.759	0.02 0.914	0.00 0.981	−0.02 0.914
Heel cushioning	R p	−0.03 0.839	−0.04 0.797	−0.01 0.941	−0.01 0.965	0.05 0.714	0.11 0.437	−0.07 0.648	0.33^*^ 0.021	−0.03 0.843	0.03 0.853	−0.07 0.611	−0.07 0.645
Forefoot cushioning	R p	−0.18 0.200	−0.19 0.185	0.18 0.198	0.11 0.434	−0.05 0.710	−0.05 0.727	0.01 0.960	0.34^*^ 0.015	0.06 0.686	0.20 0.174	0.10 0.493	0.16 0.254
Arch height	R p	−0.10 0.485	−0.10 0.478	0.11 0.437	0.02 0.883	0.11 0.431	0.18 0.199	0.15 0.294	0.31^*^ 0.029	−0.13 0.373	0.10 0.476	0.21 0.140	0.16 0.264
Mediolateral control	R p	−0.14 0.337	−0.13 0.368	0.00 0.982	−0.09 0.546	0.00 0.982	−0.01 0.930	0.01 0.957	0.06 0.683	0.23 0.107	0.21 0.151	0.02 0.868	−0.02 0.882
Overall comfort	R p	0.09 0.513	0.10 0.480	0.24 0.087	0.05 0.709	−0.01 0.958	0.02 0.864	0.03 0.840	0.16 0.257	−0.06 0.665	0.09 0.545	0.08 0.573	−0.02 0.911
Material properties of footwear	R p	0.07 0.650	0.08 0.584	0.09 0.516	0.07 0.620	0.21 0.152	0.25 0.077	0.00 0.992	0.25 0.081	−0.08 0.596	0.11 0.459	0.05 0.755	0.03 0.852

**Notes.**

L_*r*_, foot length of the right foot; L_*l*_, foot length of the left foot; W_*r*_, foot width of the right foot; W_*l*_, foot width of the left foot; Cl_*r*_, Clarke’s angle of the right foot; Cl_*l*_, Clarke’s angle of the left foot; γ_*r*_, heel angle of the right foot; γ_*l*_, heel angle of the left foot; α_*r*_, hallux valgus angle of the right foot; α_*l*_, hallux valgus angle of the left foot; β_*r*_, angle of the varus deformity of the 5th digit (β) of the right foot; β_*l*_, angle of the varus deformity of the 5th digit (β) of the left foot; R, Spearman’s rank correlation coefficient; *p*, probability value.

* α = 0.05.

In men, there was a statistically significant negative relationship of the left foot Clarke’s angle with the heel height (*R* =−0.29; *p* = 0.043), and a positive relationship between the right foot width and the arch height (*R* = 0.29; *p* = 0.044). Lower values of the Clarke’s angle were accompanied by a better perception of heel height, while higher values of the foot width correlated with a better perception of the arch height (Table 5).

**Table 5:** Relationships of foot structure features with subjective assessment of footwear comfort in men.

Variable		L_*r*_	L_*l*_	W_*r*_	W_*l*_	Cl_*r*_	Cl_*l*_	γ_*r*_	γ_*l*_	α_*r*_	α_*l*_	β_*r*_	β_*l*_
Shoe length	R p	0.04 0.756	0.02 0.866	0.12 0.402	0.07 0.648	0.01 0.971	−0.12 0.396	−0.05 0.731	0.05 0.722	0.11 0.444	0.21 0.140	0.22 0.134	0.07 0.609
Shoe forefoot width	R p	0.17 0.244	0.14 0.319	0.26 0.064	0.23 0.106	0.10 0.496	−0.07 0.638	0.06 0.669	−0.02 0.885	0.12 0.420	0.19 0.185	0.14 0.347	0.07 0.615
Shoe heel width	R p	0.04 0.804	0.04 0.785	0.12 0.413	0.14 0.331	−0.20 0.173	−0.22 0.117	−0.04 0.807	0.00 0.981	0.08 0.596	0.07 0.647	0.21 0.152	0.01 0.969
Heel height	R p	0.11 0.439	0.11 0.453	0.16 0.263	0.08 0.580	−0.21 0.151	−0.29^*^ 0.043	−0.10 0.487	−0.10 0.497	0.01 0.960	0.01 0.941	0.25 0.075	0.07 0.630
Heel cushioning	R p	−0.03 0.839	−0.02 0.875	0.16 0.253	0.15 0.286	−0.14 0.349	−0.14 0.337	0.14 0.348	0.13 0.360	0.12 0.425	0.01 0.966	0.17 0.237	−0.02 0.877
Forefoot cushioning	R p	−0.06 0.661	−0.05 0.743	0.12 0.393	0.11 0.434	−0.18 0.220	−0.17 0.242	0.03 0.863	−0.04 0.801	0.01 0.922	−0.14 0.337	0.09 0.545	0.02 0.912
Arch height	R p	0.17 0.248	0.14 0.335	0.29^*^ 0.044	0.18 0.218	−0.03 0.862	−0.12 0.420	−0.06 0.704	0.04 0.770	−0.01 0.934	−0.09 0.549	0.13 0.385	0.00 0.983
Mediolateral control	R p	−0.04 0.806	−0.07 0.654	0.08 0.591	0.02 0.891	−0.06 0.701	−0.20 0.166	−0.01 0.967	0.03 0.816	0.22 0.128	0.14 0.347	0.01 0.942	−0.06 0.692
Overall comfort	R p	0.05 0.728	0.03 0.821	0.12 0.424	0.11 0.436	0.16 0.282	0.01 0.922	−0.01 0.959	0.01 0.948	0.01 0.930	0.12 0.409	0.07 0.606	0.05 0.748
Material properties of footwear	R p	0.10 0.477	0.08 0.570	0.13 0.362	0.03 0.819	−0.10 0.501	−0.11 0.441	0.05 0.737	0.08 0.581	−0.01 0.941	−0.01 0.921	−0.01 0.938	−0.09 0.523

**Notes.**

L_*r*_, foot length of the right foot; L_*l*_, foot length of the left foot; W_*r*_, foot width of the right foot; W_*l*_, foot width of the left foot; Cl_*r*_, Clarke’s angle of the right foot; Cl_*l*_, Clarke’s angle of the left foot; γ_*r*_ , heel angle of the right foot; γ_*l*_, heel angle of the left foot; α_*r*_, hallux valgus angle of the right foot; α_*l*_, hallux valgus angle of the left foot; β_*r*_, angle of the varus deformity of the 5th digit (β) of the right foot; β_*l*_, angle of the varus deformity of the 5th digit (β) of the left foot; R, Spearman’s rank correlation coefficient; p, probability value.

* α = 0.05.

## Discussion

Our study confirmed the occurrence of dimorphic differences in the length and width of the feet in seniors, also noted by other authors (Paiva de Castro, Rebelatto & Aurichio, 2011; Saghazadeh, Kitano & Okura, 2015). In turn, the indices of longitudinal and transverse arch as well as the position of the hallux and the 5th digit did not significantly differentiate the feet of youngest-old women and men. Our study did not confirm the results of Aurichio, Rebelatto & de Castro (2011) demonstrating a greater flattening of the dynamic arch of the feet in women from São Carlos in Brazil, and Mickle et al. (2010) study of people over 60 years of age which observed greater hallux valgus in women and more varus position of the 5th digit in men. It is worth emphasizing that in both sexes, the Clarke’s angle values were lower than the normative values for the adult population, which, according to Lizis (2012), are 40–51°, while the heel angle (γ) values did not differ from the norms. The values of the hallux valgus angle (α) in both women and men were above the upper limit of the norm, for which the range of variation was assumed from 0° to 9° (Lizis, 2012), while the values of the varus angle of the Vth toe (β) ranged from 15° to 19°, which indicates their increase in relation to the norms.

An interesting and poorly investigated issue is the perception of shoe comfort, which depends on the shoe fitting to the foot and is particularly important for seniors. Piller (2006) emphasized that the feeling of comfort of the footwear as a result of its adjustment to the user’s foot is subjective and it is difficult to obtain a proper fit due to the existence of many different guidelines. According to Rossi (2001) a perfect fit of the shoes is impossible, a correct fit is highly unlikely and the only alternative remains a compromise fit. Ikpeze, Omar & Elfar (2015), López-López et al. (2016), and Menz, Auhl & Munteanu (2017) emphasized that wearing shoes that are too tight causes compression of the foot tissues, thus worsening already poor balance in the elderly. On the other hand, using shoes that are too loose causes slippage between the foot and the shoe, which in turn affects the deterioration of gait efficiency and damage to the soft tissues of the feet due to friction. In both cases, the shoes become uncomfortable for the user. Kim & Do (2019) noted that the foot length is commonly considered the most important or the only measure of footwear fit. However, according to Rossi (2001) the use of this criterion often leads to a poor fit as most of the fit problems relate to the width or volume dimension. In this study, several other footwear comfort indices were taken into account, according to Mündermann et al. (2002) and Dinato et al. (2015). Our research shows that youngest-old women, compared to men of the same age range, have a higher perception of footwear comfort for features such as the width of the shoe heel, the height of the medial arch, overall sense of comfort and material properties of the footwear. Mündermann et al. (2002) recognized that past habits and experiences could be a benchmark for assessing the footwear currently worn. Therefore, it seems that the results of the author’s own study may be dictated by the feelings resulting from the use of the shoes under evaluation. In the case of women, shoes with heels have been replaced by shoes with profiled inserts, made of materials that adjust to the feet, which result in a higher perception of comfort in terms of these features compared to men. Better perception of shoe heel width in women compared to men, as found in this study, may be the result of improved stability during shoe use, which depends *i.e.,* on the height at which the hindfoot is raised in relation to the forefoot. The studies of Tencer et al. (2004) clearly indicate that the heel height ratio to width becomes a measure of the critical angle of tipping (lateral stability). In turn, the perception of comfort in terms of the material properties of shoes can be referred to the observations of Hatton et al. (2013). They pointed out that the sensitivity of the foot sole is related to the perception of shoe comfort and the material properties of shoes, including shoe inserts. They provide plantar tactile stimulation of the central nervous system with vital information regarding the location of peak foot pressure relative to deviations from the upright body position.

The study of the relationship between footwear comfort and the shape of the foot in women showed that, the perception of footwear comfort in terms of heel cushioning, forefoot cushioning and the arch height improved as the transverse arch decreased. On the other hand, in men, the perception of footwear comfort in terms of heel height increased as the longitudinal arch decreased, and the perception of the comfort of the arch height of the shoes improved as the width of the foot increased. These data suggest that lowering the arch of the foot, as well as widening the forefoot, increases the contact surface of the foot with the shoe, hence the accompanying improvement in the perception of the comfort of its use. This seems to be confirmed by the study of Chiu & Wang (2007), who analyzed the significance of the shape of the insole and found that wearing shoes with a flat, non-profiled insole caused a reduction in the comfort of use, manifested by an increase in pain in the feet. The authors concluded that people with flat feet prefer footwear with an insole that supports the medial arch of the foot, as it increases the foot contact area with the shoe and redistributes pressure on the sole of the foot.

Summarizing the results of our study, it can be concluded that the features determining the comfort of footwear can be a useful guide in choosing footwear for women and elderly men. It is worth paying attention to the relatively high average values of comfort points, which suggest that the perception of shoe comfort is so high that this model of shoes can be recommended to seniors. The authors believe that the key element in increasing knowledge in the field in question will be the development and implementation of training programs and lectures on the selection of appropriate footwear. This information can help youngest-old men and women, their doctors and physiotherapists, as well as shop assistants in medical stores selling orthopaedic shoes and shoe designers, make the right decisions about buying and manufacturing footwear. Our study is a starting point for taking up further issues, in particular related to the aesthetic acceptability and functionality of footwear dedicated to seniors.

## Conclusions

1. Youngest-old women’s feet are shorter and narrower compared to men of the same age range. Sexual dimorphism does not concern the longitudinal arch, transverse arch, as well as the position of the hallux and the 5th digit.
2. Youngest-old women, compared to men of the same age range, have a higher perception of shoe comfort in terms of the heel width, the arch height, the overall comfort of the footwear and the material properties of the footwear.
3. In women, the perception of footwear comfort in terms of heel cushioning, forefoot cushioning, and medial arch improved as the transverse arch was lowered. On the other hand, in men, the perception of shoe comfort in terms of heel height increased as the longitudinal arch decreased, and the perception of the comfort of the arch height of the shoes improved as the foot width increased. The appropriate profile and design of the shoe allows for an increase in the contact surface of the foot with the shoe, hence the improvement of the perception of shoe comfort in people with lowered arch or with a widened forefoot.

## Supplemental Information

10.7717/peerj.12385/supp-1 Supplemental Information 1 Raw data applied for data analyses and preparation for Tables 1–5

## Additional Information and Declarations

### Data Availability

The following information was supplied regarding data availability: The raw data is available in the Supplemental File.
